# Endogenous ocular nocardiosis

**DOI:** 10.3205/oc000183

**Published:** 2021-05-17

**Authors:** George Castle, Gregory Heath

**Affiliations:** 1 York General Hospital, Ophthalmology Department, York, United Kingdom

**Keywords:** nocardia, cyriacigeorgica, ocular nocardiosis

## Abstract

Nocardiosis is an extremely rare, opportunistic, Gram-positive bacterial infection that has a high mortality rate in those patients who are immunocompromised in the presence of disseminated disease.

We describe a case of an elderly lady being treated with high-dose corticosteroids for giant cell arteritis that presented with ischaemic optic atrophy. Subsequent deterioration was accompanied by the development of subretinal lesions. Further extensive evaluation discovered she had pulmonary nocardiosis with widespread dissemination. The case has several learning points, in particular:

1. Subretinal abscesses maybe a harbinger of serious hitherto undiagnosed infection which portend a poor prognosis.
2. Vital signs in the immunocompromised may appear to be normal in the presence of serious infection.

## Introduction

Ocular infections due to nocardia are uncommon and likely to be exogenous, contracted directly through trauma, surgery, or contact lens use. Endogenous infection via the haematogenous route is very rarely reported and is usually unilateral.

This case was bilateral and is being reported here in order to help raise the clinician’s index of suspicion when subretinal abscess is seen, as the prognosis can be poor if this condition is not diagnosed rapidly, and especially in any form of immunocompromise. Time to presentation can also be delayed in the presence of any immunosuppression, and in this case the patient’s only other non-ophthalmic symptom was some weight loss. 

## Case description

The case is an 82-year-old female who was originally investigated via gastroenterology for unintentional weight loss and raised inflammatory markers, with normal CT scan of thorax, abdomen and pelvis. She was subsequently diagnosed with giant cell arteritis (biopsy positive) after she presented to the eye clinic with right arteritic ischaemic optic neuropathy and jaw claudication. She received 3 pulses of intravenous methylprednisolone only to be re-admitted 2 weeks later with what was thought to be a flare of her disease. This resulted in her receiving a further 3 pulses of the same corticosteroid.

**Figure 1:**
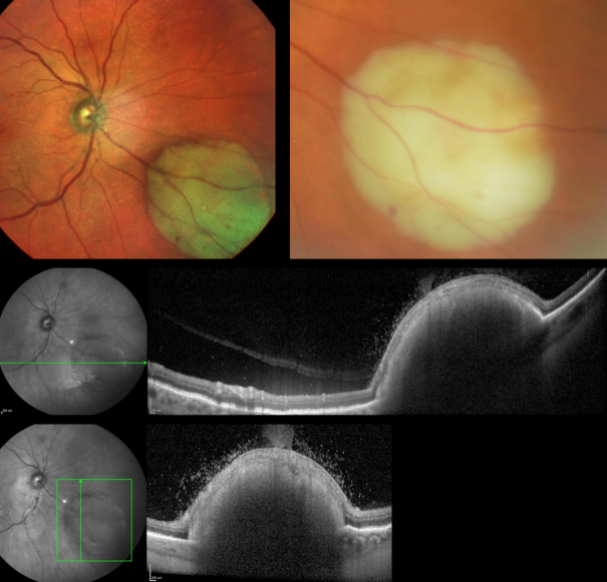
Fundal photographs and OCT scan images showing right subretinal abscesses

**Figure 2:**
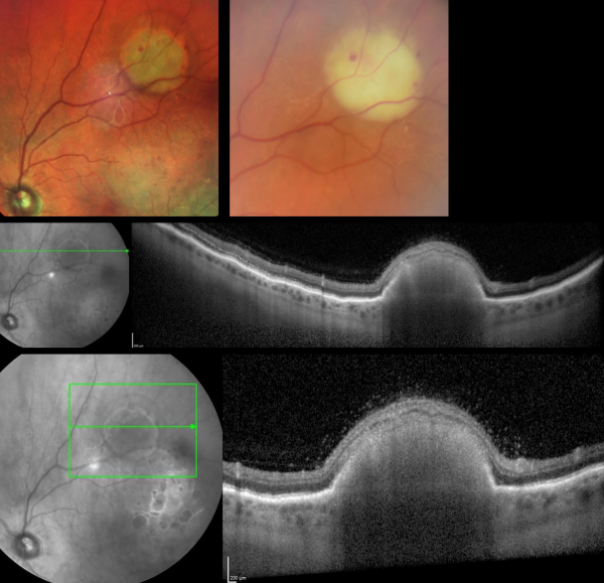
Fundal photographs and OCT scan images showing left subretinal abscesses

She was reviewed in the neuro-ophthalmology clinic 3 weeks later appearing unwell despite normal vital signs and unremarkable physical examination. Her CRP had increased and dilated ocular fundus exam revealed bilateral subretinal lesions, which were choroidal abscesses (Figure 1 (Fig. 1), Figure 2 (Fig. 2)).

**Figure 3:**
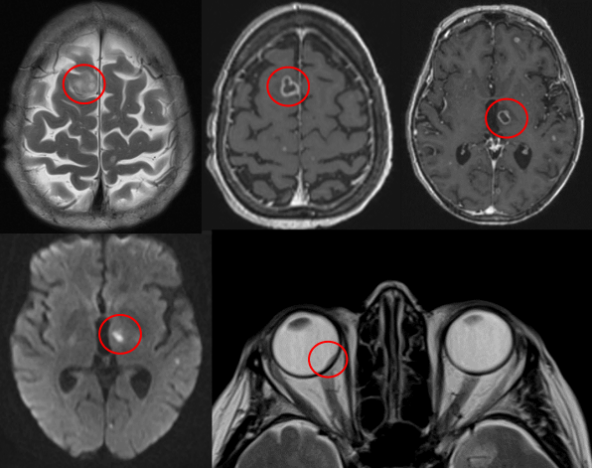
MRI brain showing disseminated cerebral nocardiosis and choroidal abscess

**Figure 4:**
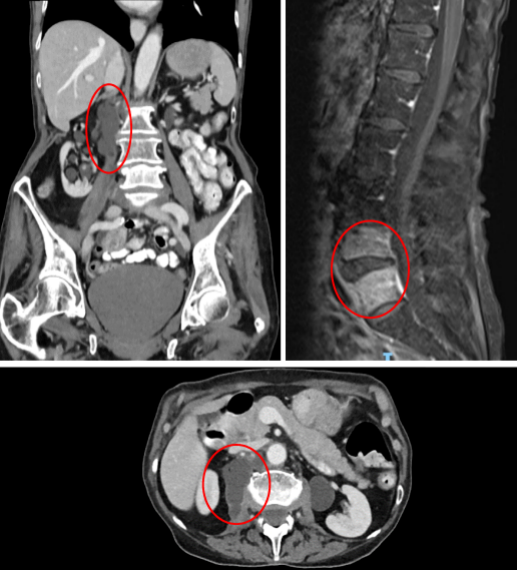
MRI scans showing psoas abscess and L4/5 discitis

She was admitted from clinic and underwent an extensive array of investigations. Three positive blood cultures confirmed that she was suffering from *Nocardia cyriacigeorgica* and imaging revealed dissemination to multiple organs. Chest x-ray now showed segmental consolidation, MRI brain showed multiple punctate lesions consistent with disseminated CNS nocardiosis; high resolution CT chest now showed scattered lung nodules, MRI spine showed appearances suggestive of a right-sided psoas abscess secondary to discitis of L4/5, and normal transoesophageal echocardiography (Figure 3 (Fig. 3), Figure 4 (Fig. 4)).

Despite receiving a course of intravenous antibiotics, there was no improvement in her general condition, and after discussion with family, treatment was subsequently withdrawn. The patient was transferred to the local hospice for palliative care where she died.

## Discussion

The genus of bacteria *Nocardia* was named after a French microbiologist Edmund Nocard, describing veterinary infections in the nineteenth century. This opportunistic Gram-positive bacterium causes human infection mainly in the immunocompromised. The most common species that can infect humans are found in the soil, and when they cause infections, they are mostly self-limiting, unless in the immunocompromised.

Ocular *Nocardia* infections are more often exogenous, such as corneal infection after eye trauma, but endogenous cases spread by the haematogenous route usually present as choroidal abscess in immunocompromised patients with pneumonia-like symptoms. Making the diagnosis can be challenging, so treatment might be delayed, and this case displays that there may be very little in the way of physical signs.

It is felt that 50% of pulmonary *Nocardia* infections disseminate in the bloodstream, particularly intracranial lesions [1]. Disseminated nocardiosis is an overwhelming disease with high mortality. Indeed, some cases are diagnosed at autopsy [2].

Endogenous ocular deposits of *Nocardia* are still very rare, and in fact barely 40 cases have been reported in the literature in the past 50 years [3]. It is widely held that the earlier the diagnosis is made, the greater the chance of patient recovery [4]. The later the diagnosis, the worse the visual prognosis [5]. Therefore, *Nocardia* must be included in the differential diagnosis of patients that present with signs of intraocular infection, who are immunosuppressed [6]. *Nocardia *endophthalmitis can occur in the immunocompetent but is less common [7]. Diagnosis can be very difficult due to the variation in symptoms and severity at presentation [8].

## Conclusion

In our immunosuppressed patient, her vital signs were normal for a long time in the face of severe underlying infection. Her choroidal abscesses were the first sign of her underlying problem, and so a particularly high index of suspicion is needed in immunosuppressed patients in view of the high mortality rate.

Learning points in this case included that subretinal abscesses may be a harbinger of serious hitherto undiagnosed infection which portend a poor prognosis. Furthermore, it must be noted that vital signs in the immunocompromised may appear to be normal in the presence of serious infection.

## Notes

### Competing interests

The authors declare that they have no competing interests.

## References

[1] Gregor RJ, Chong CA, Augsburger JJ, Eagle RC, Carlson KM, Jessup M, Wong S, Naids R (1989). Endogenous Nocardia asteroides subretinal abscess diagnosed by transvitreal fine-needle aspiration biopsy. Retina.

[2] Rogers SJ, Johnson BL (1977). Endogenous Nocardia endophthalmitis: report of a case in a patient treated for lymphocytic lymphoma. Ann Ophthalmol.

[3] Eschle-Meniconi ME, Guex-Crosier Y, Wolfensberger TJ (2011). Endogenous ocular nocardiosis: an interventional case report with a review of the literature. Surv Ophthalmol.

[4] Phillips WB, Shields CL, Shields JA, Eagle RC, Masciulli L, Yarian DL (1992). Nocardia choroidal abscess. Br J Ophthalmol.

[5] Trehan H, Kaushik J, Jain VK, Parihar JK, Avasthi A (2017). Endogenous Nocardial Endophthalmitis in an Immunosuppressed Patient: A Serious Warning of an Underlying Life Threatening and Blinding Disorder. J Ophthalmic Vis Res.

[6] Silva RA, Young R, Sridhar J, Nocardia Study Group (2015). Nocardia Choroidal Abscess: Risk Factors, Treatment Strategies, and Visual Outcomes. Retina.

[7] Dave VP, Pathengay A, Sharma S, Naveen N, Basu S, Pappuru RR, Das T (2019). Diagnosis, Clinical Presentations, and Outcomes of Nocardia Endophthalmitis. Am J Ophthalmol.

[8] Puri S, Hadayer A, Breaux A, Barr CC (2018). Disseminated Nocardiosis with retinal abscess in a patient treated for bullous pemphigoid. Am J Ophthalmol Case Rep.

